# Author Correction: Lead halide perovskite vortex microlasers

**DOI:** 10.1038/s41467-021-23079-y

**Published:** 2021-04-30

**Authors:** Wenzhao Sun, Yilin Liu, Geyang Qu, Yubin Fan, Wei Dai, Yuhan Wang, Qinghai Song, Jiecai Han, Shumin Xiao

**Affiliations:** 1 State Key Laboratory on Tunable laser Technology, Ministry of Industry and Information Technology Key Lab of Micro-Nano Optoelectronic Information System, Shenzhen Graduate School, Harbin Institute of Technology, 518055 Shenzhen, PR China; 2 Collaborative Innovation Center of Extreme Optics, Shanxi University, Taiyuan, 030006 Shanxi PR China; 3 National Key Laboratory of Science and Technology on Advanced Composites in Special Environments, Harbin Institute of Technology, 150080 Harbin, PR China

**Keywords:** Lasers, LEDs and light sources, Organic-inorganic nanostructures

Correction to: *Nature Communications* 10.1038/s41467-020-18669-1, published online 25 September 2020.

The original version of this Article omitted Yilin Liu as an equally contributing author. This has now been corrected in both the PDF and HTML versions of the Article.

