# Supplementary material for: Lead halide perovskite vortex microlasers
Source: Nat Commun. 2020 Sep 25;11:4862. doi: 10.1038/s41467-020-18669-1 (PMC7519163; doi:10.1038/s41467-020-18669-1)
Supplement: Supplementary file 1 — Supplemental Information [file 41467_2020_18669_MOESM1_ESM.pdf]

Supplementary Information for

Lead halide perovskite vortex microlasers

Wenzhao Sun[1,†], Yilin Liu[1,†], Geyang Qu[1], Yubin Fan[1], Wei Dai[1], Yuhan Wang[1], Qinghai Song[1,2], Jiecai Han[3], Shumin Xiao[1,2,3,*]

[1.] State Key Laboratory on Tunable laser Technology, Ministry of Industry and Information Technology Key Lab of Micro-Nano Optoelectronic Information System, Shenzhen Graduate School, Harbin Institute of Technology, Shenzhen, 518055, P. R. China.

[2.] Collaborative Innovation Center of Extreme Optics, Shanxi University, Taiyuan, 030006, Shanxi, P. R. China.

[3.] National Key Laboratory of Science and Technology on Advanced Composites in Special Environments, Harbin Institute of Technology, Harbin 150080, P. R. China.

Email: *shumin.xiao@hit.edu.cn

In the main text, we have reported the realization of vortex microlasers with different topological charge. In this supplementary information, we show the experimental details about the perovskite films, the fabrication technique, the numerical simulation, the optical characterization of vortex microlasers.

Contents:

Supplementary Methods

Supplementary Figure 1-27

Supplementary Discussion

Supplementary Methods

The characterization of lead halide perovskite films. The $MAPbBr_3$ perovskite thin film is prepared by the spin coating method. Because the chlorobenzene solution can effectively fill the gaps between the perovskite grains and prevent the formation of large particle crystals, the perovskite thin film is very dense. It can be seen from the atomic force microscope (AFM) image in Supplementary Figure 1. In the area of 25×25 μm, no obvious voids can be seen and the roughness mean square is 6.15 nm. These characteristics are good enough for optoelectronic applications.

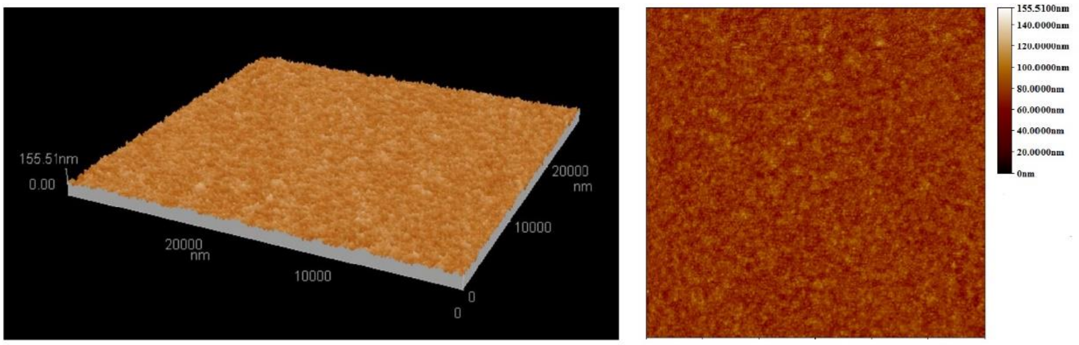

Supplementary Figure 1. The AFM image of the $MAPbXBr_3$ film.

Then the material properties of the sin-coated film were characterized. Supplementary Figure 2 shows the Energy-dispersive X-ray spectroscopy (EDS) results of the films. It can be clearly seen that the ratio of Br/Pb is 74.14:25.86≈ 2.867. This ratio is close to 3 and indicates the formation of $MAPbBr_3$ film.

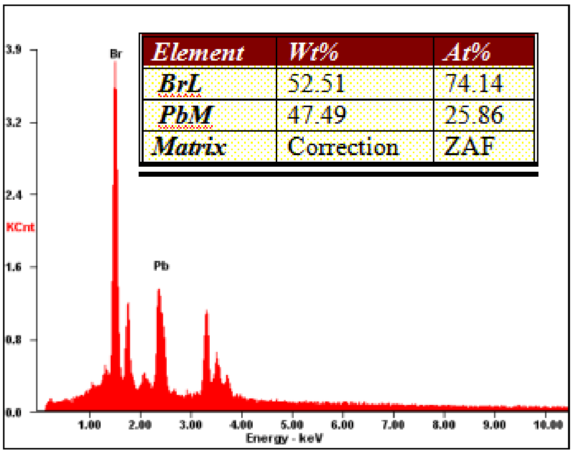

Supplementary Figure 2. EDS of the $MAPbXBr_3$ film

Then the X-ray diffraction spectrum of the synthesized film were recorded. Four peaks at 14.85°、30.05°、45.85°and 62.55° can be clearly seen. These peaks can be indexed to the (001), (002), (003) and (004). Both of the above observations of EDS and XRD are consistent with the previous reports. Therefore, we can confirm the formation of high-quality $MAPbBr_3$ perovskite film, which is the basis of the whole research.

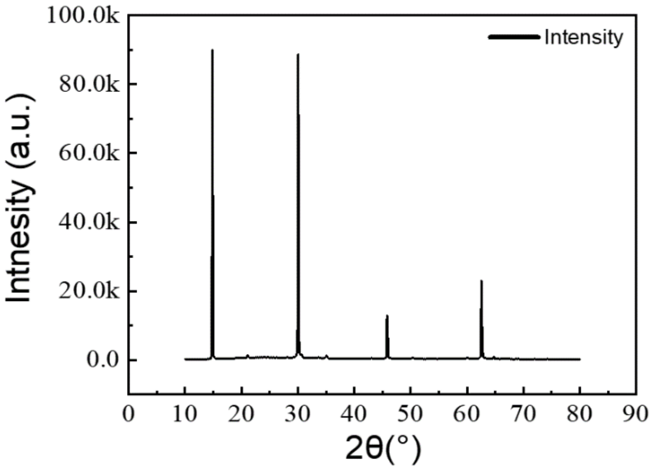

Supplementary Figure 3. XRD of the $MAPbXBr_3$ film

We measured the refractive index of the 300 nm perovskite film by an ellipsometer, and the results are shown in the Figure S4

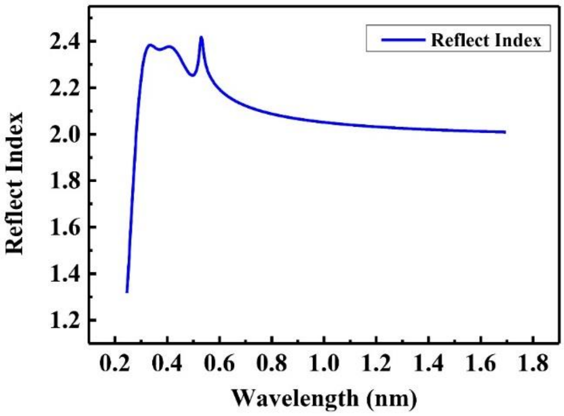

Supplementary Figure 4. The reflective index of the 300nm perovskite film as a function of wavelength.

The etching process of $MAPbBr_3$ films. In the main text, we have mentioned that the etching proces of $MAPbBr_3$. Here we show the experimental details to identify the high quality of the etching process. In Figure 5 of the main text we have already shown the top-view scanning electron microscope (SEM). Here we show the cross-section SEM image to show the selectivity and the etching rate.

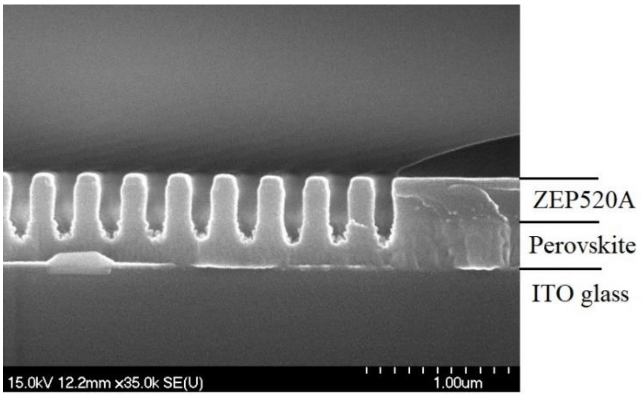

Supplementary Figure 5. The cross-section SEM image of perovskite grating.

Supplementary Figure 5 shows the side-view SEM image of one grating. The sample was etched with the mixture of H_2 and Ar in an inductively coupled plasma etcher. From the SEM image, we can see that the $MAPbBr_3$ was removed very clean during the etching process. The sidewall is quite straight and the tilt angle is around 79 degree. After measuring the reduction of the thickness of ZEP520A and the depth of $MAPbBr_3$ gap. It is easy to know the selectivity between ZEP520A and $MAPbBr_3$ are around 1:3. The above information is important to achieve high quality perovskite nanostructures.

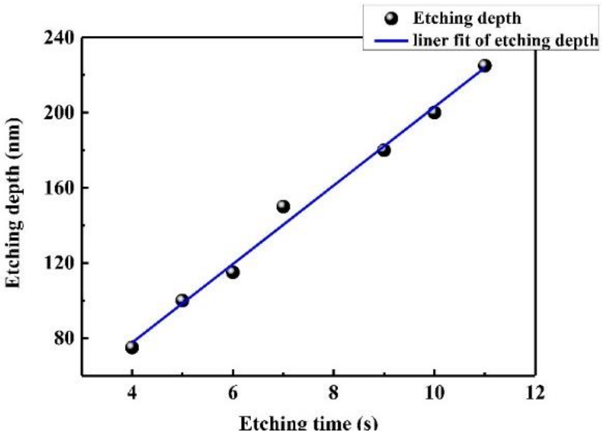

Supplementary Figure 6. The etching rate of the new recipe.

By repeating the etching process for many rounds, we have measured the etching depth with the side-view SEM images and plotted the data as a function of etching time. It is easy to see that the etching depth is linearly dependent on the time with an etching rate of 21 nm/s. This etching rate has quite good repeatability and thus provide an approach to precisely control the etching depth. This is essential to acquire shallow etching samples that are frequently used in the photonic integrated circuits.

Since the etching process plays an essential role in the manuscript, the comparison of photoluminescence before and after the etching is very important and necessary. We have compared the photoluminescence spectra from a grating and a film with the same pump fluence. The results are summarized in Figure S7. It is easy to see that the central peak positions of two spectra are very close. The intensity of photoluminescence from the grating is about 1477.

Considering the air region in the grating (~ 0.225), the scaled intensity should be around 1477/0.775=1905, which is close to the measured value from the film (1935). The slight difference should be induced by the intensity variation of the pump laser. Therefore, we can confirm that the etching process won't degrade the photoluminescence of lead halide perovskites.

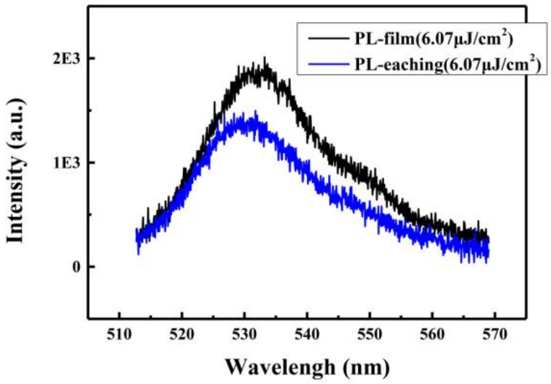

Supplementary Figure 7. The emission spectra from a grating (blue line) and a film (black line) under the same pump fluence.

The lasing actions in conventional perovskite grating. In the main text, we have mentioned two advantages of this etching recipe. The good controllability in etching depth has been shown in the side-view SEM of the gratings. Here we demonstrate the other properties. As the Cl_2 has been replaced by the H_2/Ar_2 in ICP etching, the ion exchange process between Cl^- and Br^- is supposed to be suppressed. The additional conversion process within low pressure chemical vapor deposition can be saved. Therefore, both of the nanostructures and its gain characteristics can be preserved in nanostructures. With the same process as the main text and Fig. S4, we have fabricated a perovskite grating with period (p) and gap size (g) of 282 nm and 84 nm, respectively. The etching depth is also around 200 nm.

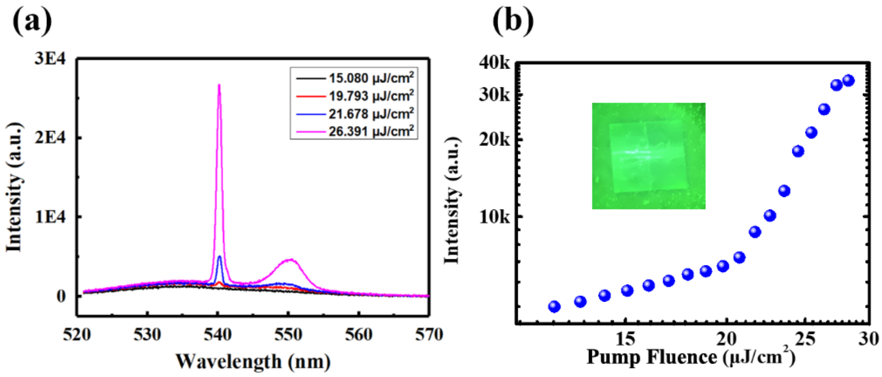

Supplementary Figure 8. Laser action of perovskite grating. (a) The top-view SEM image of the perovskite grating (b) The evolution of single-mode emission laser spectrum. (c) The output intensity (solid line) as a function of pumping density.

Then the perovskite grating was optically pumped under a home-made optical microscope

system. The experimental results are summarized in Fig. S6. With the increase of pump power, we can also see the transition from a broad spontaneous emission to a single mode peak at 540 nm. Supplementary Figure 8 (b) shows the dependence of integrated output intensity around 540 nm on the pumping density. It is easy to see the obvious S-shaped curve. The corresponding slopes are ~ 1, 3.56, and 1. All of these observations are consistent with the main text and clearly show the onset of perovskite microlasers.

Numerical simulations of $MAPbBr_3$ Bullseye microlasers. In the main text, we have shown the simulation of $MAPbBr_3$ bullseye grating. Here we show more details about the far field pattern and the resonant modes. With all the same parameters as the main text, we can see that the mode is confined around the central defect in the waveguide. This is the reason that we select shallow etching. It is much easier to support a waveguide mode in the radial direction. The right panel shows the corresponding far field pattern. Owing to the rotation symmetry of bullseye, the emission in left panel forms a donut at the center.

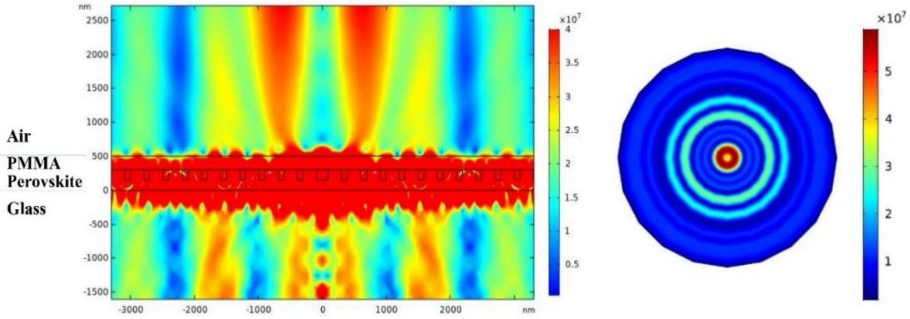

Supplementary Figure 9. Numerical simulation eigenmode and schematic of the cross section.

The shape of beam profile and the direction of laser emission can be tuned by controlling the gratings in bullseyes. Supplementary Figure 9 shows the resonance mode field diagrams of grating periods with 270 nm, 275 nm and 280 nm, respectively. And the resonant wavelength is at 540.71 nm, 541.03 nm and 540.47 nm, respectively. The other parameters are the same as the main text. In the Supplementary Figure 10 we can learn that the grating period affects both of the emission directionality and the divergence angle of perovskite microlaser. In this sense, the structural parameters must be optimized to maintain the vertical cavity surface emitting lasers (VCSELs).

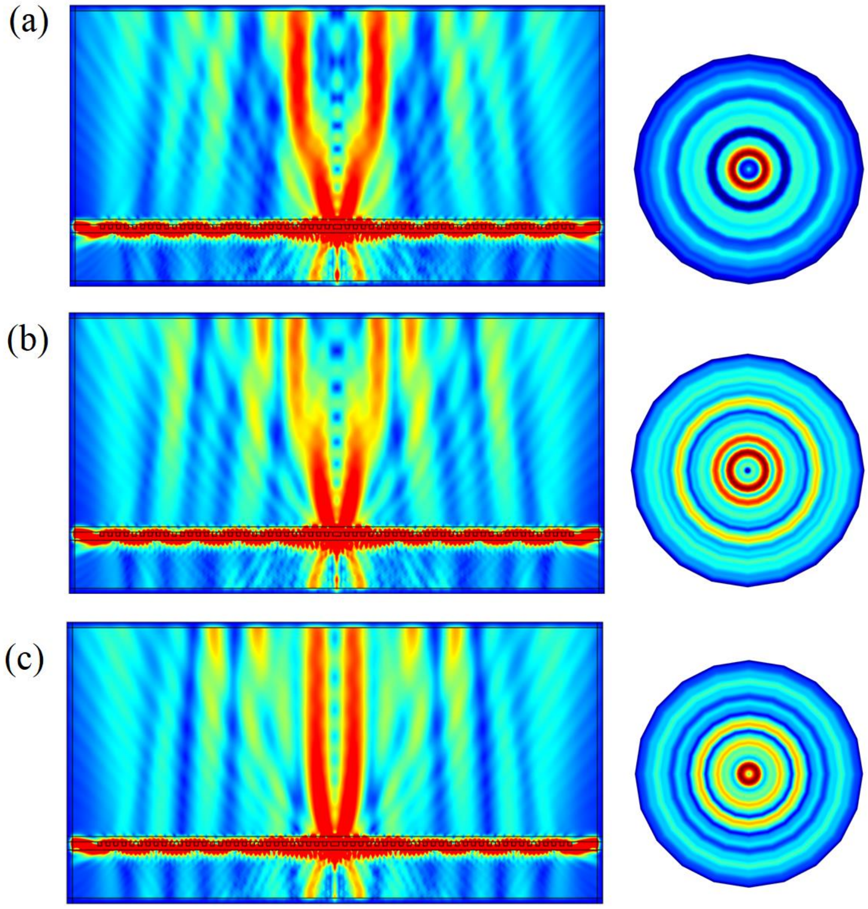

Supplementary Figure 10. Numerical simulation eigenmode and schematic of the cross section. (a) the resonance mode field diagrams with grating periods of 270nm (b) the resonance mode field diagrams with grating periods of 275nm (c) the resonance mode field diagrams with grating periods of 280nm

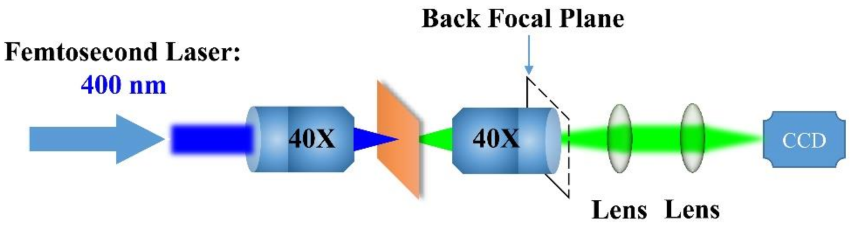

Supplementary Figure 11. Back focal plane imaging equipment setup

The optical setups measuring vortex lasers. In Figure 2(a) of the main text, the back focal plane image is shown. Here we show the experimental details to ahcieve this back focal iamge. The experimental equipment used is such a set as shown in Supplementary Figure 11. The 400 nm femtosecond laser was focused to the sample plane through a 40× objective lens. By adjusting the second 40x objective lens, the imaging information of the Fourier object plane

is mapped to the back focal plane imaging system

The slef-interference pattern is esential to determine the orbital angular momentum. The experimental details are shown in Supplementary Figure 12. The frequency doubled femtosecond laser was focused onto the sample by a 40× objective lens. The perovksite laser emissions is collected and colliminated to a hgihly directional laser beam. The laser emission is split into two beams. One beam propagates directly and the other beam pass the delay line. By adjusting the delay line, two beams match again and produces the self-interference pattern. For the donut beam, two beams are laterally shifted. The bright ring of one beam overlaps the dark center of the other one.

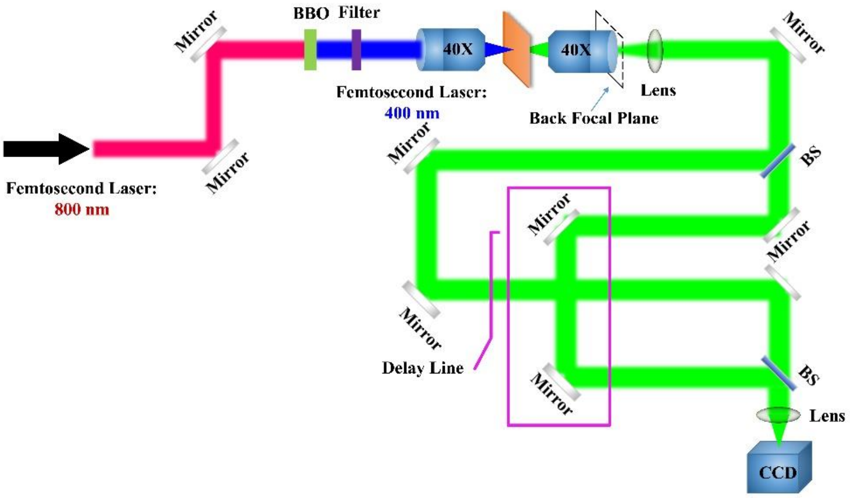

Supplementary Figure 12. The setup for self-interference experiment.

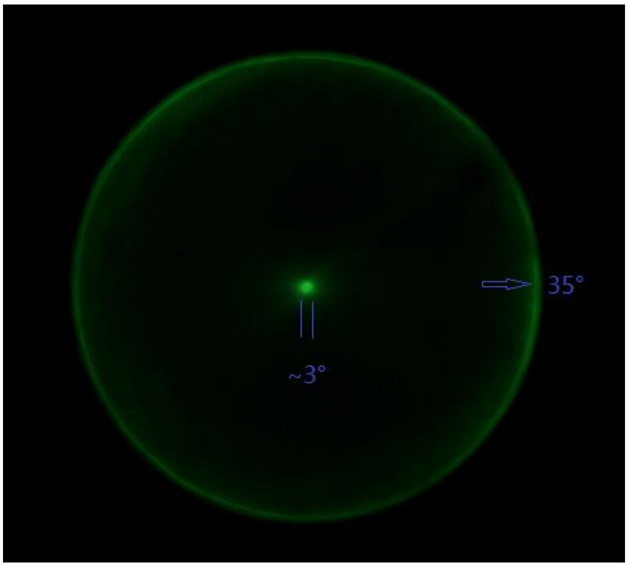

Supplementary Figure 13. The wide angle far field distribution of perovskite VCSELs.

Supplementary Figure 13 shows the wide angle far field distribution of the perovskite VCSELs. The donut beams in the main text appears at the center of this figure. The large green circle corresponds to the maximal collection angle of the objective lens, which is 35 degree. As

a result, the divergent angle of the center donut can be determined at ~ 3°. This is a direct proof of the highly directional output of the perovskite VCSELs.

In the main text, we have also found that the divergent angle increases with the increase of topological charge. This phenomenon is understandable. In principle, the angular distribution of a vortex beam can be expressed as

$$u_p^q(r,\theta) = \sqrt{\frac{2p!}{\pi(|q|+p)!}}\frac{1}{w_0}\left(\frac{\sqrt{2}r}{w_0}\right)^{|q|} \times \exp\left(-\frac{r^2}{w_0^2}\right)\exp\left(iq\theta\right) \tag{1}$$

From the formula (1), u is the complex amplitude of the light field, it is clear to see that the field dependent on the topological charge q. We therefore calculated the far field angular distributions of the vortex beam with different topological charge. As shown in Supplementary Figure 14 below, the dark center gets larger at large topological charge, consistent with our experimental observations.

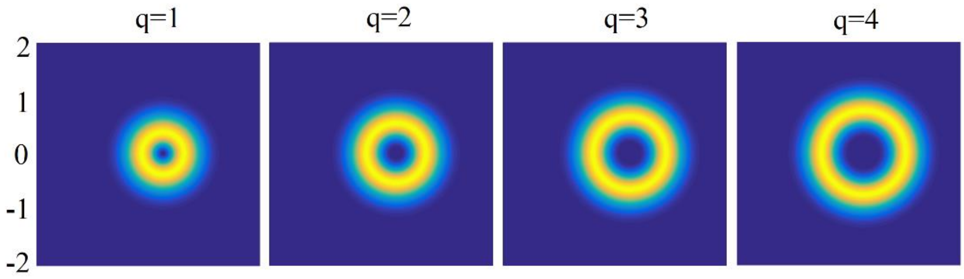

Supplementary Figure 14. Field pattern of vortex beam under different q values

The optical characterization of vortex lasers. We have studied the pump profile on the grating. As depicted in Figure S15, the beam size is about 10 μm, which is smaller than the grating size and locates at the center of the grating. Considering the lattice size of the grating, the pump beam roughly covers 36 periods of the grating.

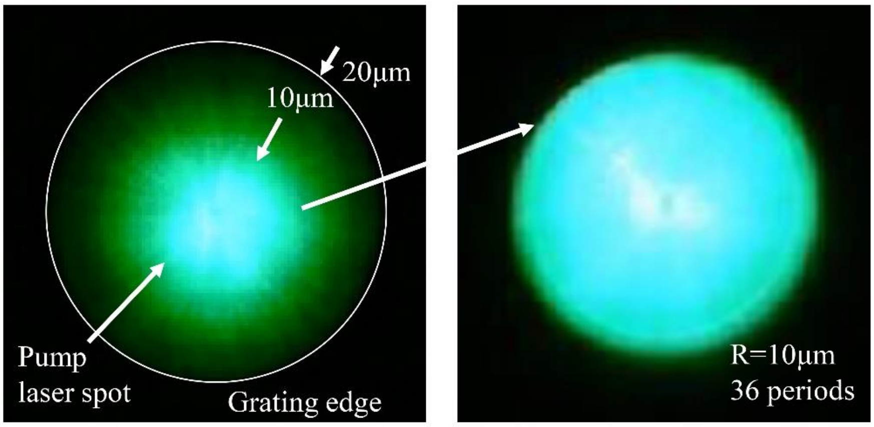

Supplementary Figure 15. The pump profile on the grating. The beam size is about 10 μm, which is smaller than the grating size.

Supplementary Figure 15 shows the profile of pump laser on the perovskite spiral grating. The beam size is about 10 μm, which is smaller than the overall size of grating. According to

the lattice size of the grating, the gain area roughly covers 36 periods

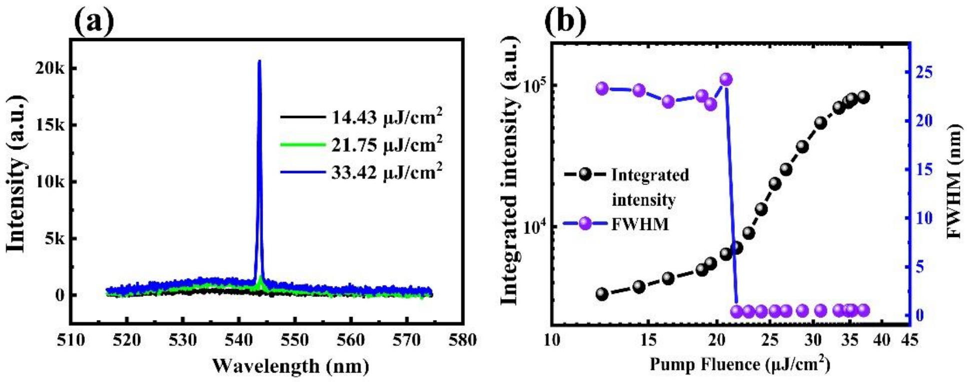

Supplementary Figure 16. (a) The laser spectra at different pumping fluence from the Archimedean grating with arm number *l = 2*. (b) The integrated output intensities (black dots) and the linewidth (purple dots) as a function of pump fluence.

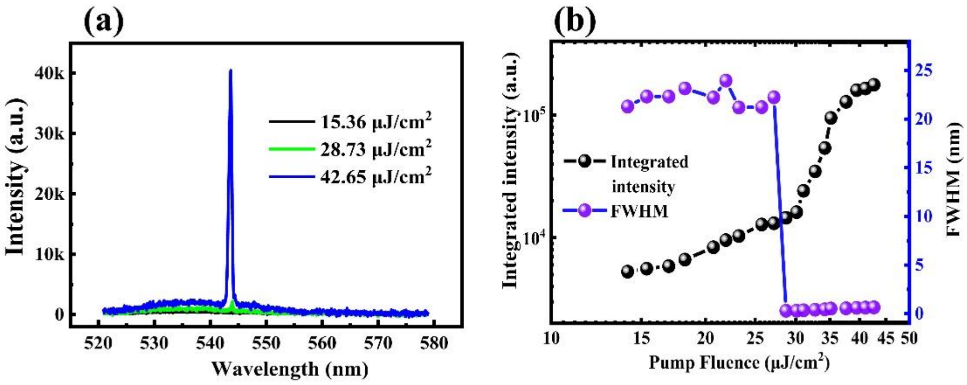

Supplementary Figure 17. (a) The laser spectra at different pumping fluence from the Archimedean grating with arm number l = 3. (b) The integrated output intensities (black dots) and the linewidth (purple dots) as a function of pump fluence.

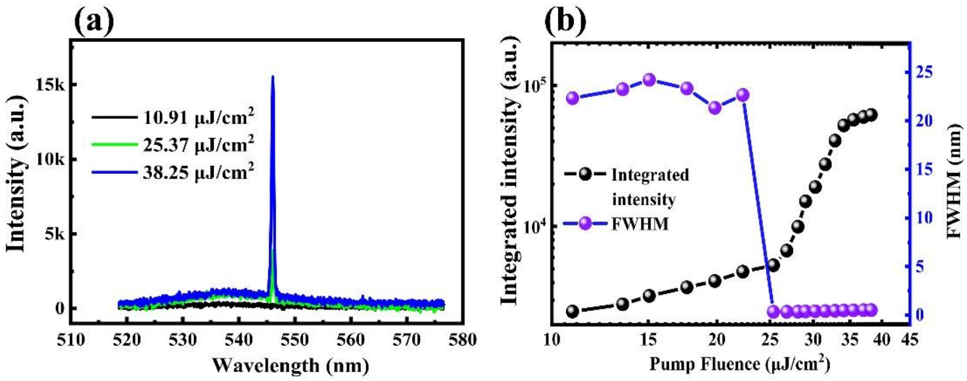

Supplementary Figure 18. (a) The laser spectra at different pumping fluence from the Archimedean grating with arm number *l = 4*. (b) The integrated output intensities (black dots) and the linewidth (purple dots) as a function of pump fluence.

In the main text, we have also shown the far field donut shapes of vortex beams with different topological charge |q| = 2-4. Here we show the corresponding laser spectrum and thresholds. All the results are summarized in Supplementary Figure 16-18. With the increase of pumping density, it is easy to see that the spontaneous emission gradually transits to a single mode around 546 nm. The corresponding threshold curves also give a clear S shape in log field. The transition in spectrum, the S curve, and the reduction of linewidth all confirm the onset of lasing actions in Archimedean spirals with different arm numbers.

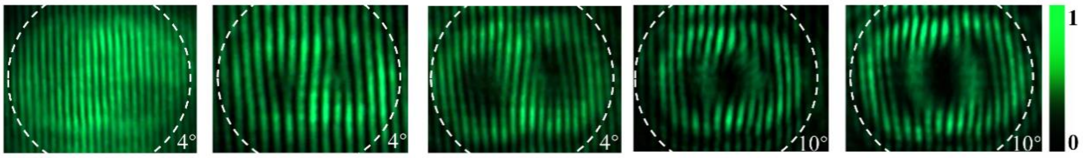

Supplementary Figure 19. The self-interference patterns of emission beams in the backward direction with L= 0 ~ -4

We have also mentioned the interference pattern of large topological charge. The results in forward direction are plotted in the main text. Here we show the results in the backward direction. As shown in Supplementary Figure 19, there is no folks in bullseye VCSEL. With the increase of arm number from -1~-4, there are 1, 2, 3, and 4 sets of inverted forks， consistent with the theoretical expectation well.

All orders of our vortex laser can maintain single-mode even at saturation threshold. The laser spectra given above are all at the saturation threshold. The emission peak energy is constant as a function of the input power density. Taking the laser with q=0 as an example, we measured two sets of laser spectra and thresholds around one day apart. It can be seen from the Supplementary Figure 20 that the laser energy is still stable, and the threshold curves are similar.

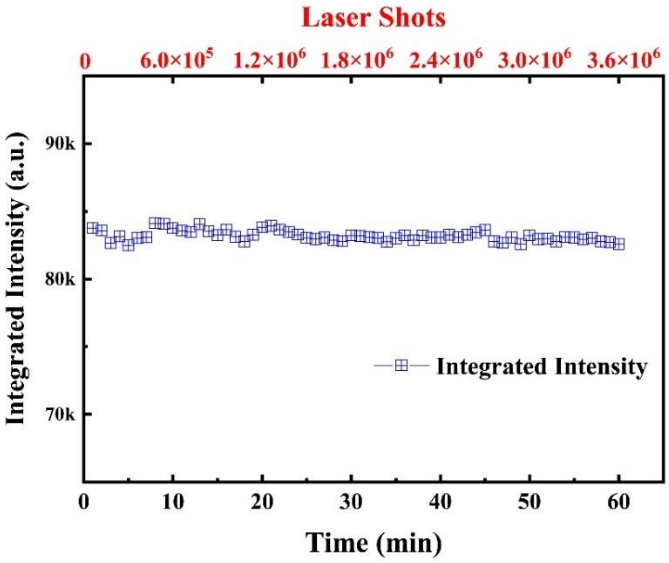

Supplementary Figure 20. The stability of our perovskite microlasers.

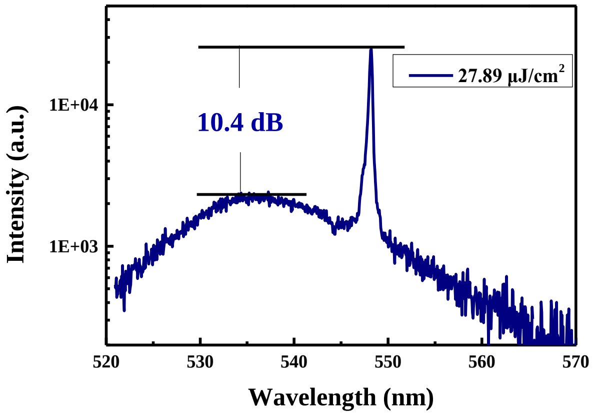

Supplementary Figure 21: The intensity difference between the laser peak and the spontaneous emission background.

As shown in Supplementary Figure 21 below, the intensity difference can be above 10 dB between the laser peak and the spontaneous emission background. It can prove that the laser has reached saturation.

Supplementary Figures

High order vortex lasers with q=8, 16 and 32. As the topological charge number increases, due to the limitations of micro-nano fabrication accuracy of microstructures, vortex lasers are more difficult to obtain. In our revised manuscript, we successfully implemented OAM vortex lasers with q=8, 16 and 32. The result is as shown in Supplementary Figure 22.

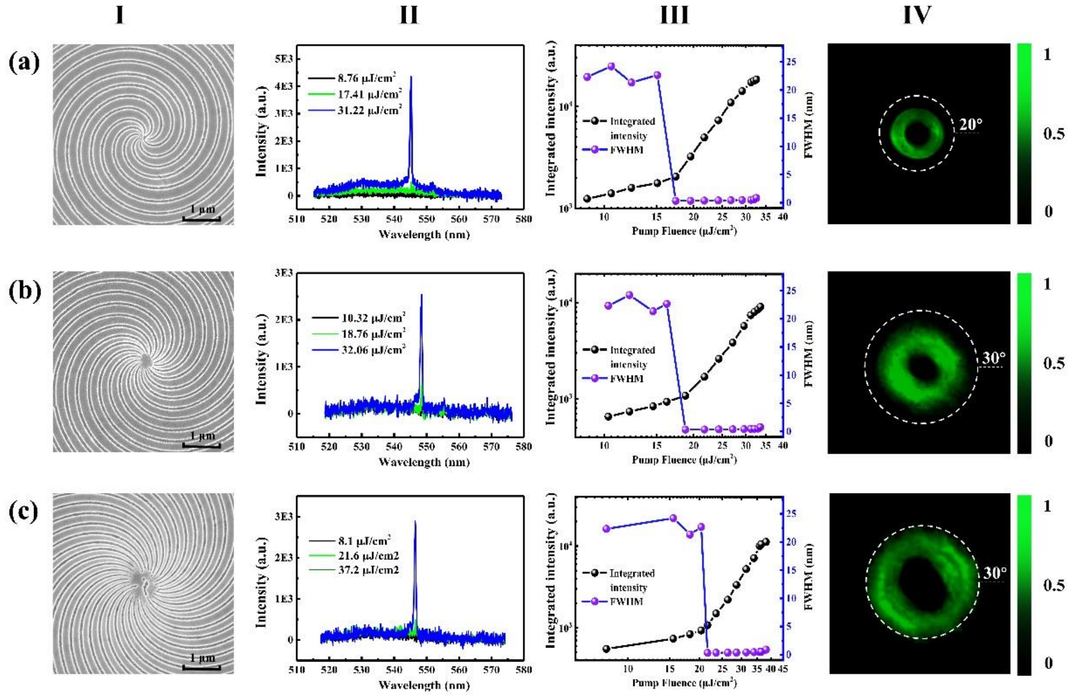

Supplementary Figure 22. The vortex laser emissions from Archimedean spirals with large arm number of l = 8 (a), 16 (b), and 32 (c). Columns-I, II, III, and IV show the top-view SEM images, the laser spectra at different pump fluence, threshold behaviors, and the back focal plane images of the vortex lasers.

It can be seen that when q=8, there is still no adhesion at the center of the structure. When q=16, the central area with a size of 200 nm is stuck. When q=32, adhesion occurs in the central area with a size of 700nm. However, the size of these adhesion areas is relatively small compared to the grating structure. We are still able to get single mode laser and ring spot. Supplementary Figure 23 (a), (b) and (c) shows the back focal plane polarization imaging with q=8, 16 and 32.

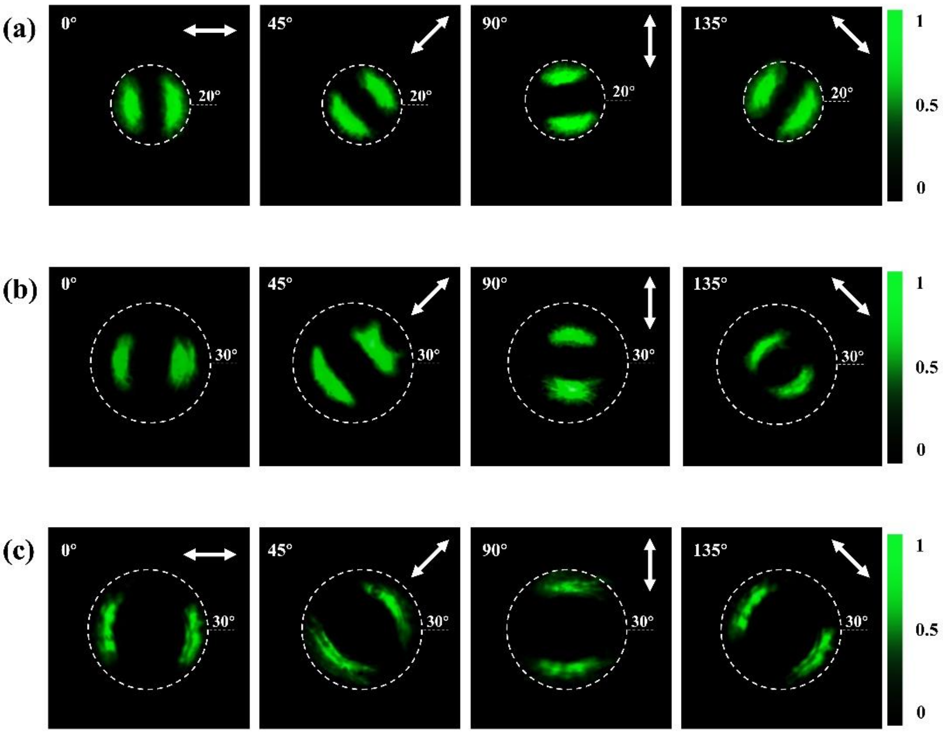

Supplementary Figure 23. The polarization states of the donut beams from Archimedean spirals with arm number of l = 8 (a), 16 (b), and 32 (c). From left column to the right column, the polarization axis is 0°, 45°, 90°, and 135°, respectively.

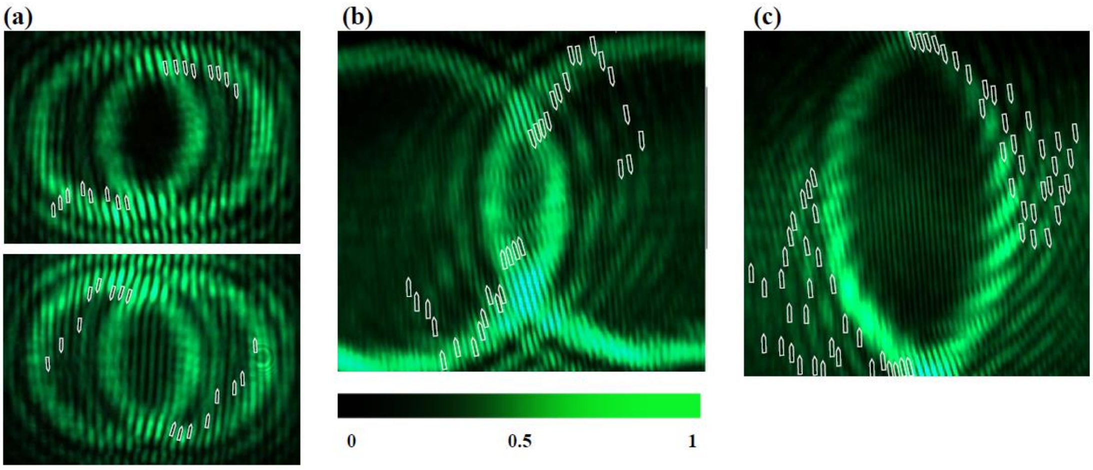

Supplementary Figure 24. The self-coherent interference patterns of Archimedean spirals with arm number of l = 8 (a), 16 (b), and 32 (c).

It is meaningful that we can also verify the properties of the vortex laser at q=8, 16, 32 by self-coherent interference. The results are shown in the Supplementary Figure 24. It can be seen from the self-coherent interference picture that there are 8, 16, and 32 pairs of forks, respectively. It is proved that we have obtained 8, 16, and 32 topological charge vortex lasers. The perovskite spiral grating method used in this manuscript can achieve high-order topological charge vortex lasers.

Supplementary Discussion

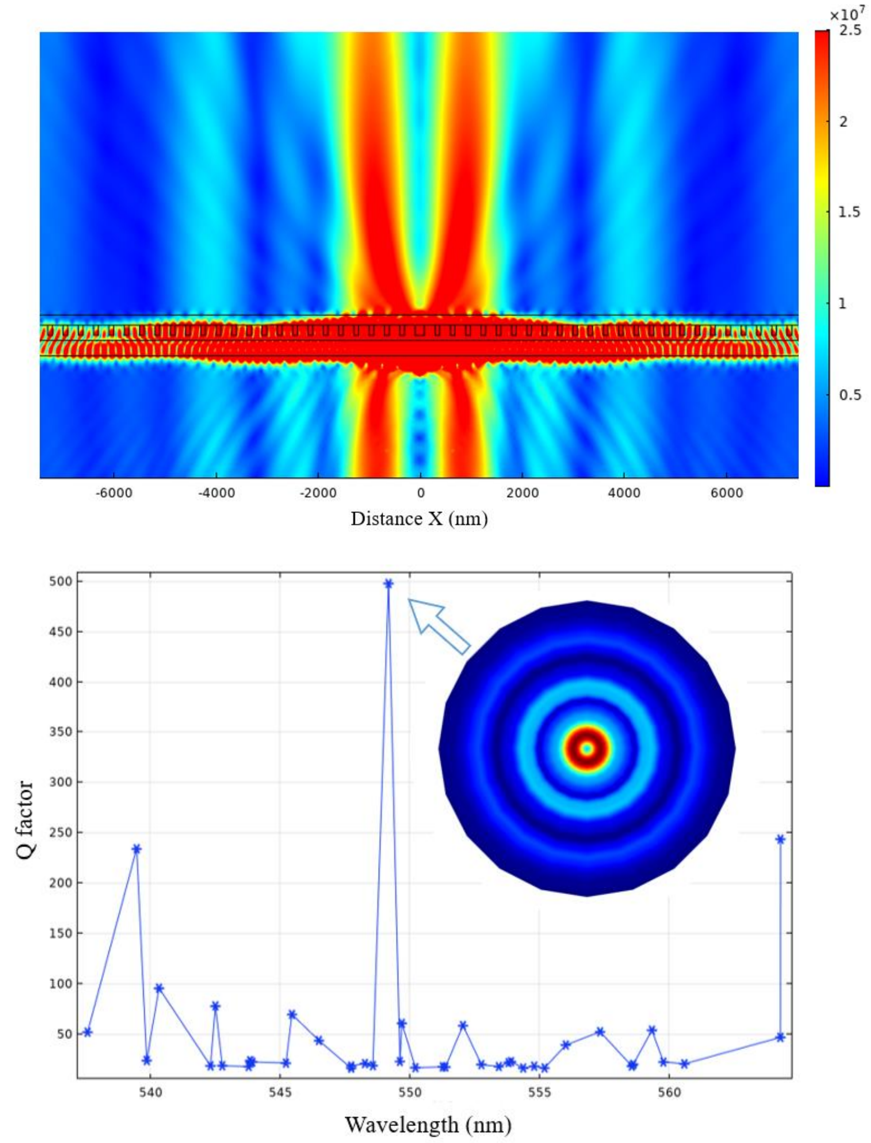

Supplementary Figure 25. Bullseye VCSEL simulation of perovskite on silicon nitride substrate

Bullseye grating on Si_3N_4 substrate. One important question is whether the same design works on a high refractive index substrate. This is essential for the application in hybrid photonic integrated circuits. For some designs such as photonic crystals, this is not possible due to the strong leakage to the substrate. For the designed grating, owing to the shallow etching, it is still possible to form the VCSELs with different topological charges. To extend the potential application of our research, we have replaced the ITO glass substrate with a commercial silicon nitride substrate. There is 300 nm Si_3N_4 layer on the top of a 3 μm SiO_2 insulating layer. The thickness of perovskite film is still 300 nm and shallow etched 200 nm. The simulation result is plotted in Fig. S17. The Si_3N_4 layer can function as part of the waveguide. By adjusting the

parameters, the period p is 295 nm and the fill factor f is 0.7, the other parameters are the same as the main text, a VCSEL mode with the highest Q factor and donut beam profile can still be achieved. This clearly confirms that the design in our research holds true for high index substrate and this is one intrinsic difference between our design and the previous reports.

The degree of radial polarization of the beam. We found that the purity of the lasing mode radial polarization in our structure was limited by the symmetry of the structure. It can be seen from the Figure 2 in the main text that the polarized spot shape is two donuts. The previous experimental sample had a symmetry defect, which caused the spots to be misaligned, the light spots are misaligned and overlap with each other, resulting in the deformation of the light spots. In the manuscript, it has been replaced with a new polarization diagram which was measured with new sample.

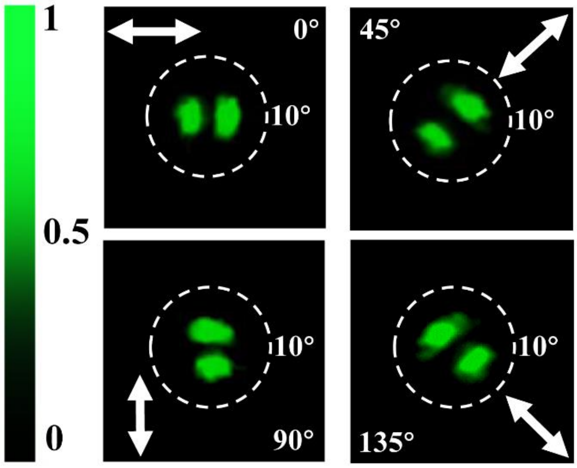

Supplementary Figure 26. The radial polarization of VCSEL with q = 0. After passing a linear polarizer, the donut beam turns into two separated lobes along the direction of linear polarizer.

We want to declare that the vortex laser generated in our symmetric structure is radially polarized which was proved by experiment. However, due to fabrication deviation and edge roughness it will still cause a small circular polarization component. And this circular polarization component is accidental, the angle between the linear polarization of the emission and the polarizer axis appears randomly within ±5 degrees.
