# Supplementary material for: Lead halide perovskite vortex microlasers
Source: Nat Commun. 2020 Sep 25;11:4862. doi: 10.1038/s41467-020-18669-1 (PMC7519163; doi:10.1038/s41467-020-18669-1)
Supplement: Supplementary file 3 — Lasing Reporting Summary [file 41467_2020_18669_MOESM3_ESM.pdf]

nature research

Corresponding author(s): Shumin Xiao

Lasing Reporting Summary

Nature Research wishes to improve the reproducibility of the work that we publish. This form is intended for publication with all accepted papers reporting claims of lasing and provides structure for consistency and transparency in reporting. Some list items might not apply to an individual manuscript, but all fields must be completed for clarity.

For further information on Nature Research policies, including our data availability policy, see Authors & Referees.

Experimental design

Please check: are the following details reported in the manuscript?

1. Threshold

Plots of device output power versus pump power over a wide range of values indicating a clear threshold

☒ Yes ☐ No — Figure 1,3 in the manuscript

2. Linewidth narrowing

Plots of spectral power density for the emission at pump powers below, around, and above the lasing threshold, indicating a clear linewidth narrowing at threshold

☒ Yes ☐ No — Figure 1,3 in the manuscript

Resolution of the spectrometer used to make spectral measurements

☒ Yes ☐ No — Figure 1,3 in the manuscript

3. Coherent emission

Measurements of the coherence and/or polarization of the emission

☒ Yes ☐ No — Figure 2,4 and 5 in the manuscript

4. Beam spatial profile

Image and/or measurement of the spatial shape and profile of the emission, showing a well-defined beam above threshold

☒ Yes ☐ No — Figure 2,3 and 5 in the manuscript

5. Operating conditions

Description of the laser and pumping conditions
Continuous-wave, pulsed, temperature of operation

☒ Yes ☐ No — Femtosecond pulsed laser

Threshold values provided as density values (e.g. W cm^{-2} or J cm^{-2}) taking into account the area of the device

☒ Yes ☐ No — 21 μJ/cm2

6. Alternative explanations

Reasoning as to why alternative explanations have been ruled out as responsible for the emission characteristics
e.g. amplified spontaneous, directional scattering; modification of fluorescence spectrum by the cavity

☒ Yes ☐ No — Para 1,3 in Page 6 in the manuscript, Para 1,2 in Page 10 in the manuscript

7. Theoretical analysis

Theoretical analysis that ensures that the experimental values measured are realistic and reasonable
e.g. laser threshold, linewidth, cavity gain-loss, efficiency

☒ Yes ☐ No — Section1, 3 in Supplementary information

8. Statistics

Number of devices fabricated and tested

☒ Yes ☐ No — More than ten sets of samples

Statistical analysis of the device performance and lifetime (time to failure)

☒ Yes ☐ No — Able to keep the laser stable after 3600000 pulses,and the sample can maintain good laser performance for nearly two weeks after packaging

March 2018
